# Chromatin-associated APC regulates gene expression in collaboration with canonical WNT signaling and AP-1

**DOI:** 10.18632/oncotarget.25781

**Published:** 2018-07-27

**Authors:** William Hankey, Zhong Chen, Maxwell J. Bergman, Max O. Fernandez, Baris Hancioglu, Xun Lan, Anil G. Jegga, Jie Zhang, Victor X. Jin, Bruce J. Aronow, Qianben Wang, Joanna Groden

**Affiliations:** ^1^ Department of Cancer Biology and Genetics, The Ohio State University College of Medicine, Columbus, Ohio, United States of America; ^2^ Department of Pathology, Duke University School of Medicine, Durham, North Carolina, United States of America; ^3^ Biomedical Informatics Shared Resource, The Ohio State University, Columbus, Ohio, United States of America; ^4^ Department of Basic Medical Sciences, Tsinghua University School of Medicine, Beijing, China; ^5^ Division of Bioinformatics, Cincinnati Children's Hospital Medical Center, Cincinnati, Ohio, United States of America; ^6^ Department of Medical and Molecular Genetics, Indiana University School of Medicine, Indianapolis, Indiana, United States of America; ^7^ Department of Molecular Medicine, University of Texas Health Science Center at San Antonio, San Antonio, Texas, United States of America

**Keywords:** APC, AP-1, canonical WNT signaling, chromatin, colorectal cancer

## Abstract

Mutation of the *APC* gene occurs in a high percentage of colorectal tumors and is a central event driving tumor initiation in the large intestine. The APC protein performs multiple tumor suppressor functions including negative regulation of the canonical WNT signaling pathway by both cytoplasmic and nuclear mechanisms. Published reports that APC interacts with β-catenin in the chromatin fraction to repress WNT-activated targets have raised the possibility that chromatin-associated APC participates more broadly in mechanisms of transcriptional control. This screening study has used chromatin immunoprecipitation and next-generation sequencing to identify APC-associated genomic regions in colon cancer cell lines. Initial target selection was performed by comparison and statistical analysis of 3,985 genomic regions associated with the APC protein to whole transcriptome sequencing data from APC-deficient and APC-wild-type colon cancer cells, and two types of murine colon adenomas characterized by activated Wnt signaling.

289 transcripts altered in expression following APC loss in human cells were linked to APC-associated genomic regions. High-confidence targets additionally validated in mouse adenomas included 16 increased and 9 decreased in expression following APC loss, indicating that chromatin-associated APC may antagonize canonical WNT signaling at both WNT-activated and WNT-repressed targets. Motif analysis and comparison to ChIP-seq datasets for other transcription factors identified a prevalence of binding sites for the TCF7L2 and AP-1 transcription factors in APC-associated genomic regions. Our results indicate that canonical WNT signaling can collaborate with or antagonize the AP-1 transcription factor to fine-tune the expression of shared target genes in the colorectal epithelium. Future therapeutic strategies for APC-deficient colorectal cancers might be expanded to include agents targeting the AP-1 pathway.

## INTRODUCTION

Biallelic *APC* mutations initiate the development of a high percentage of colorectal cancers [1, 2]. A*PC* encodes a multi-purpose protein whose functions include negative regulation of the canonical WNT signaling pathway [3]. The APC protein inactivates canonical WNT signaling by limiting availability of β-catenin [4], a licensing factor that modifies how members of the TCF/LEF family of transcription factors regulate gene transcription [5]. APC interacts with β-catenin in a cytoplasmic complex that facilitates β-catenin degradation [6–8], while nuclear APC facilitates both β-catenin export to the cytoplasm [9–11] and β-catenin removal from specific genomic loci [12]. Interaction of APC with chromatin-associated β-catenin negatively regulates the expression of *MYC*, *AXIN2*, *DKK1* and SP5 [12, 13], four known WNT targets.

The contribution of APC loss to gene expression has been assumed to be exclusively β-catenin-mediated, although this has not been broadly addressed experimentally. This study was designed to identify a more comprehensive list of genes transcriptionally regulated by chromatin-associated APC and to determine whether or not APC mediates their transcriptional repression exclusively through displacement of β-catenin from TCF/LEF family transcription factor complexes.

Chromatin immunoprecipitation of APC and next-generation sequencing were performed from HCT-116 colon cancer cells, which express wild-type APC yet can model the APC loss observed in the majority of colorectal cancers following transient siRNA-based silencing. Gene expression data were collected from HCT-116 cells in the presence or absence of *siRNA* targeting *APC* and were compared to ChIP-seq data to identify candidate genes controlled by chromatin-associated APC. High-confidence candidate genes were likely shared targets of canonical WNT signaling and surprisingly included both genes increased in expression following APC loss and decreased in expression following APC loss.

APC-associated genomic sequences identified in our initial screening step exhibited enrichment of validated transcription factor binding sites for both TCF7L2 and AP-1, and co-occurrence of these transcription factors within many of these same genomic regions. These results indicate that AP-1 modulation should be investigated as a potential therapeutic strategy for targeting the expression of a large subset of canonical WNT target genes.

## RESULTS

### APC ChIP-seq identified 3,985 APC-associated genomic regions

**Figure 1:**
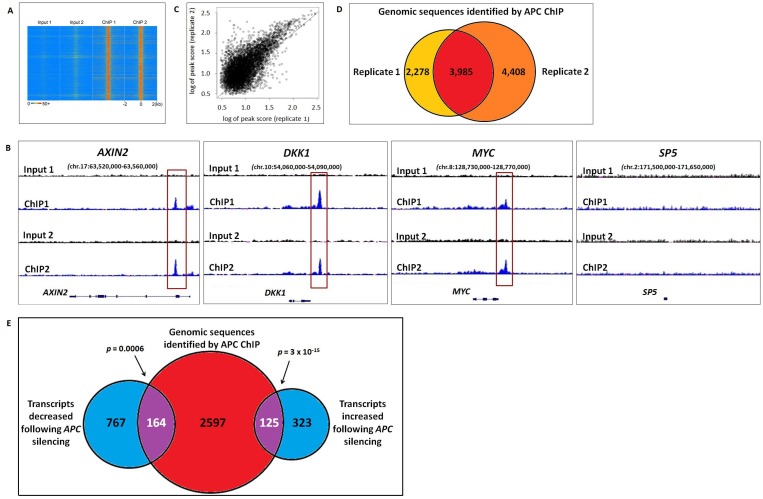
Genomic sequences enriched by APC ChIP overlap with transcripts altered in expression following APC loss (**A**) A heat map with rows corresponding to 4-kb genomic regions centered on each peak shared between replicates 1 and 2 shows high ChIP signal intensity (red) in each ChIP-seq sample and low background (blue) in each matching input sample. (**B**) Sequencing data visualized using the Integrative Genomic Viewer confirm that three out of four positive control loci (*AXIN2*, *DKK1*, *MYC* and *SP5*) known to be regulated by chromatin-associated APC include genomic regions (red boxes) enriched by APC ChIP. (**C**) A scatter plot compares peak scores (in log scale) from ChIP-seq replicates 1 (x-axis) and 2 (y-axis), indicating high signal:background ratio and consistency in peaks scores (Pearson correlation coefficient *ρ* = 0.8246). (**D**) Genomic peaks identified in both APC ChIP-seq replicates (*p* < 0.00001) were defined as overlapping if their summits were separated by less than 400-bp (the median of peak width in the peak calling results). (**E**) 2,886 genes associated with one or more genomic sequences enriched in both APC ChIP-seq replicates were compared to 448 transcripts that increased and 931 transcripts that decreased in the same cell line following *APC* silencing (*q* < 0.05). 289 overlapping genes (purple) were identified as potential targets of direct transcriptional control by chromatin-associated APC.

Chromatin immunoprecipitation of APC was performed in two biological replicates from HCT-116 colon cancer cells that express wild-type APC but also a degradation-resistant point mutant of β-catenin that constitutively activates canonical WNT signaling. Previously published reports identified four genes (*MYC*, *AXIN2*, *SP5* and *DKK1*) regulated by chromatin-associated APC [12, 13], and peak-calling thresholds were adjusted to pass the stringent false discovery rate (FDR) cutoff of 1.07% (Figure 1A) while retaining peaks at three out of four of these internal positive control loci (Figure 1B). ChIP-seq data quality was consistent between replicates 1 and 2 (Figure 1C). 3,985 genomic regions enriched by APC ChIP (*p* < 0.00001) were identified in both replicates relative to their respective inputs (Figure 1D).

### RNA-seq data identify 289 APC-responsive transcripts encoded by genes located near genomic regions enriched by APC ChIP-seq

**Figure 2:**
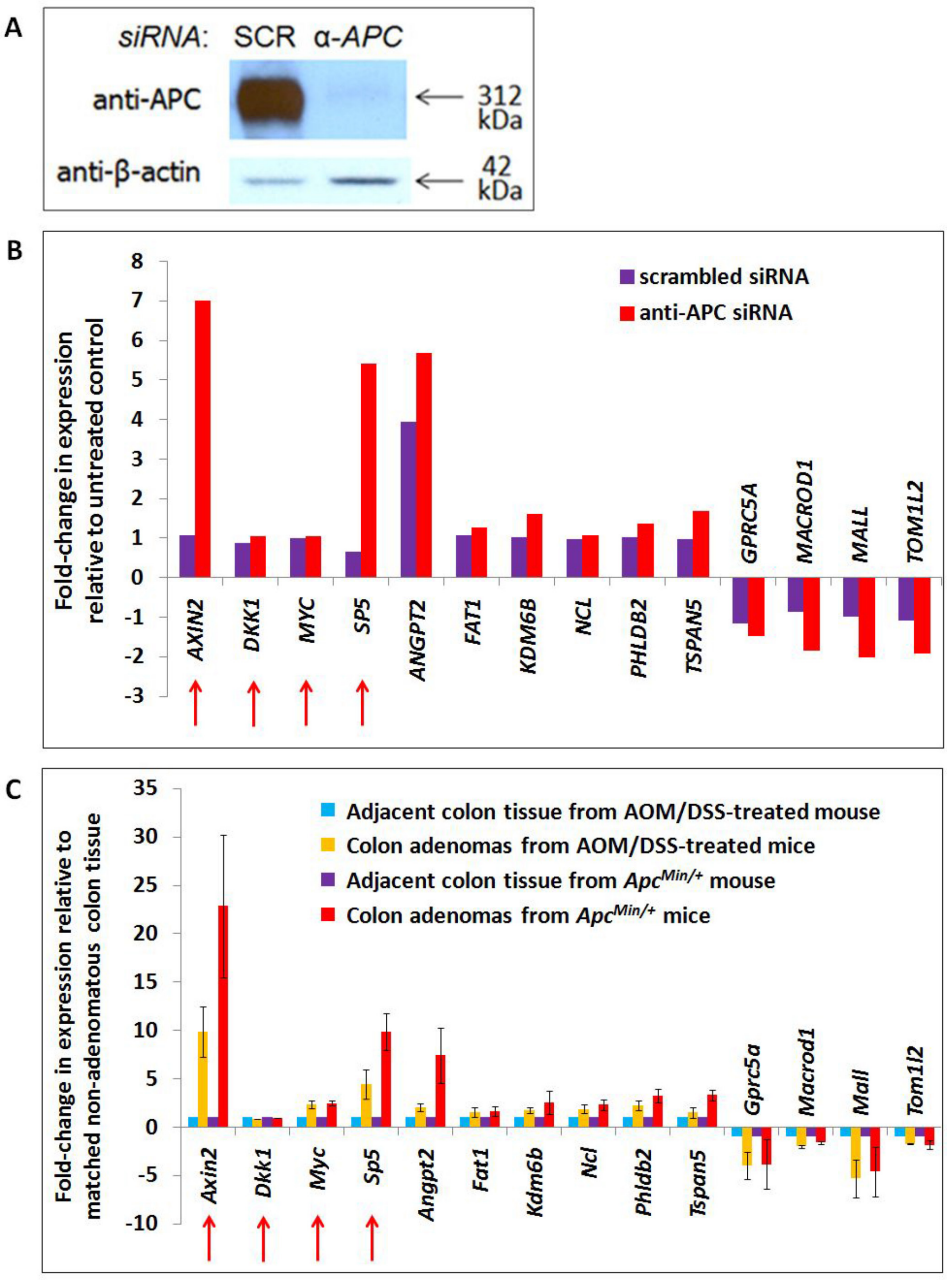
Transcription of APC-associated genes changes in APC-deficient cells and mouse adenomas with activated WNT signaling A list of 2,886 genes associated with genomic regions enriched by APC ChIP were compared with 1,379 transcripts altered in expression following *APC* silencing by *siRNA* in the same cell line (Panels **A**, **B**) and 1,535 transcripts altered in expression following both *Apc* loss and *Ctnnb1* activation in mouse colon adenomas (Panel **C**). Forty-nine genes satisfied both screening criteria, including ten candidates depicted in Panels B and C, and two positive controls (*AXIN2* and *SP5*, indicated with red arrows). Two other positive controls (also indicated with red arrows) failed to satisfy these screening criteria due to an insignificant change in gene expression in either the cell line model alone (*MYC*) or in both models (*DKK1*). RNA-seq data for these genes are included in Panels B (human cell line) and C (mouse adenomas). Error bars representing standard deviation are included only for conditions for which multiple samples were sequenced.

The effects of chromatin-associated APC binding on gene transcription were evaluated by RNA-seq analysis of HCT-116 cells in the presence or absence of *siRNA* reducing *APC* expression. Differential expression analysis using Cufflinks software [14] identified 1,379 transcripts altered in expression following *APC* silencing (*q* < 0.05), including two out of four positive control transcripts (*AXIN2* and *SP5*, Figure 2B). Transcripts were compared to 3,985 genomic sequences enriched in both APC ChIP-seq replicates, which had been assigned to 2,886 different genes based on proximity to transcription start sites. Comparison of the ChIP-seq and RNA-seq analyses identified 125 genes increased and 164 genes decreased in expression following *APC* silencing (Figure 1E). This indicates that chromatin-associated APC may act as a transcriptional repressor or activator depending on the chromatin context, despite the fact that chromatin-associated APC antagonizes canonical WNT activation of all four target genes identified in previous studies [12, 13].

### Mouse tumor RNA-seq identifies some APC-sensitive transcripts as targets of canonical Wnt signaling

HCT-116 cells are mismatch repair-deficient and relatively unstable. To circumvent these limitations and select candidate targets shared with *in vivo* models of tumorigenesis, genes of interest were filtered further using colon adenoma RNA-seq data from two mouse models of colon tumorigenesis on a *C57BL/6J* background [15]: one expressing wild-type Apc and a degradation-resistant mutant β-catenin (as a result of treatment with azoxymethane and dextran sulfate sodium (AOM/DSS-treated) [16]) and the other with loss of function mutations of Apc (*Apc^Min/+^*) (Figure 1E). These two models of canonical Wnt-driven mouse intestinal tumorigenesis were selected for their abilities to generate tumors in the colon specifically, resulting in a better basis for comparison to the HCT-116 colon cancer cell model. The use of two adenoma types enabled identification of shared (canonical Wnt-driven) transcriptional changes with higher confidence while filtering out confounding factors (such as the inflammatory component of the AOM/DSS model) [15, 17]. While they differ in that adenomas from the *Apc^Min/+^* model lack functional Apc whereas those from AOM/DSS-treated mice retain wild-type Apc and its chromatin-associated functions, loss of the Apc-dependent cytoplasmic mechanism promoting β-catenin degradation is a common feature of both adenoma types that makes them useful for the identification of canonical Wnt target genes. Of the 289 candidate transcripts from the previous filtering step, 49 changed in expression in the same direction as in the human RNA-seq data by at least 1.5-fold in both *Apc^Min/+^* and AOM/DSS colon adenomas relative to non-adenoma colon tissue controls (by one-sample *t*-test with a significance cutoff of FDR < 0.1). These represent candidate target genes altered by canonical WNT signaling. 31 genes increased while 18 genes decreased in *APC/Apc*-deficient cells and adenomas. An additional filtering criterion focused on potential transcriptional regulatory elements identified by APC ChIP-seq and located within 10 kb upstream of transcription start sites or within first introns. This further reduced the number of candidate genes to 16 increased and 9 decreased following APC loss (siRNA-based, shown in Figure 2A). Ten candidate genes (Figure 2B, 2C) were selected for further study based on APC ChIP-qPCR validation of enrichment comparable to positive control peaks. APC ChIP enrichment of genomic regions in these ten loci relative to alpha-satellite repeat negative controls and comparable to *AXIN2* intron 1 and *MYC* promoter element positive controls is clearly visible by qPCR in Supplementary Figure 3B (although the overall purpose of the figure is different). Expression values for these ten targets are depicted from the mouse (Figure 2C) and human (Figure 2B) RNA-seq datasets.

### Binding sites for AP-1 and several promoter-associated transcription factors were enriched in APC peaks

**Figure 3:**
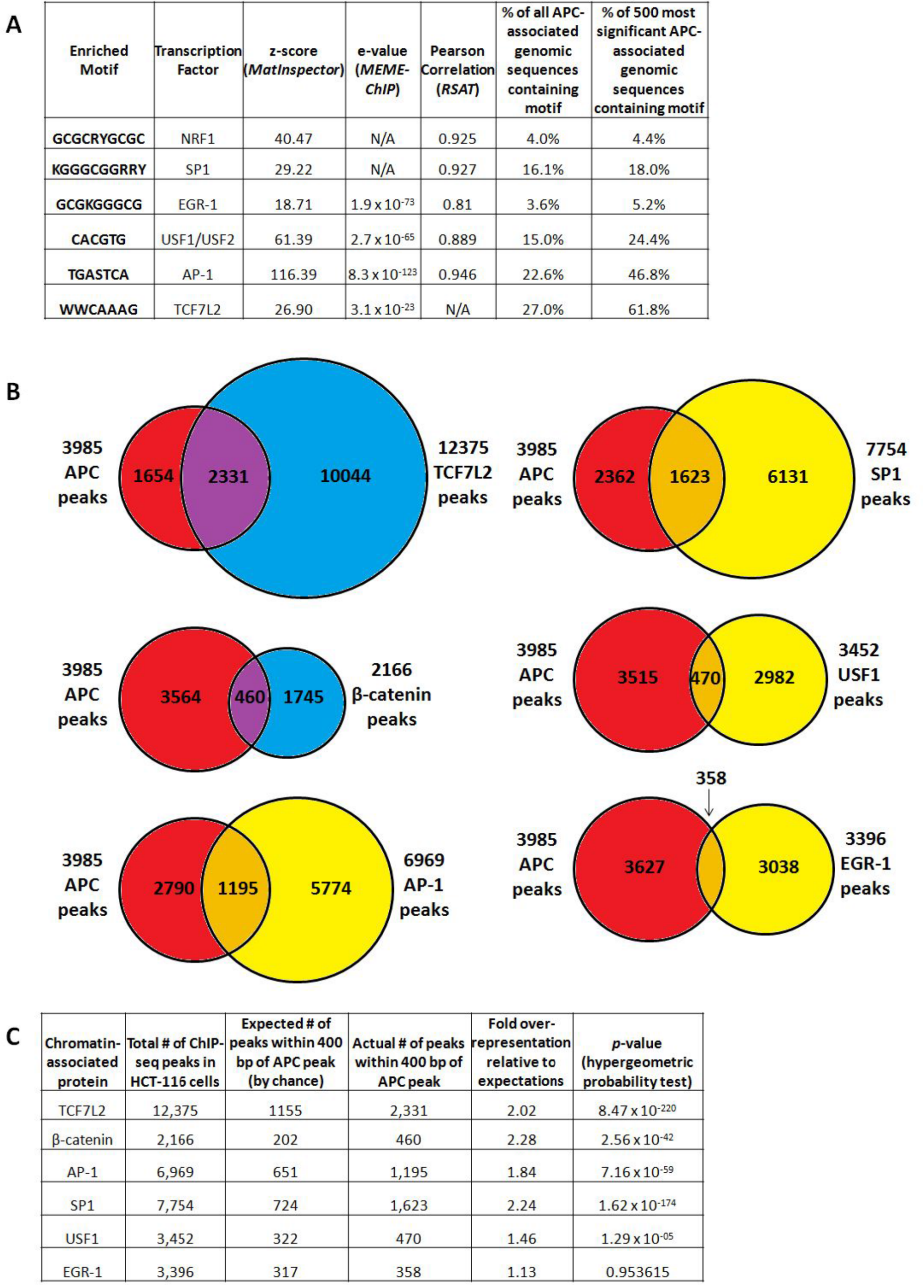
APC-associated genomic sequences are enriched for TCF7L2, AP-1 and SP1 transcription factor binding motifs (**A**) Motif analysis of all peaks using the *MatInspector* (Genomatix Software Suite), *MEME-ChIP* and *Regulatory Sequence Analysis Tools* (*RSAT*) algorithms detected significant enrichment of predicted binding sites, particularly for the AP-1, EGR-1 and USF1/USF2 transcription factors. All transcription factor binding motifs occurred more frequently in the subset of 500 genomic sequences enriched by APC ChIP with the lowest *p*-values, particularly those for TCF7L2 (as expected) and AP-1. (**B**) 3,985 APC ChIP-seq peaks were compared with corresponding peaks from several transcription factor ChIP-seq experiments. (**C**) Overlap data were tabulated to calculate fold overrepresentation of each transcription factor binding site among APC-associated genomic regions, relative to expected background (*p*-values calculated by hypergeometric probability test).

We asked whether chromatin-associated APC is recruited to specific genomic sequences exclusively through a mechanism mediated by interaction with β-catenin [12] or through multiple mechanisms. Sequences enriched by APC ChIP were subjected to motif analysis using the *MatInspector* (from the Genomatix Software Suite) [18], *MEME-ChIP* [19] and *Regulatory Sequence Analysis Tools* (*RSAT*) [20] algorithms (Figure 3A). All three algorithms detected enrichment of binding sites for the AP-1 transcription factor (variations of the TPA response element, TGASTCA), while TCF7L2 binding sites (expected to predominate based on the association of APC with β-catenin and its transcription factor-binding partner TCF7L2) were significantly enriched according to *MEME-ChIP* and *MatInspector* only (Figure 3A). Enriched motifs identified by multiple algorithms (TCF7L2, AP-1, NRF1, SP1, EGR-1 and USF1/2 binding sites) were further investigated as candidates to mediate APC recruitment and transcriptional control.

### The highest-confidence peak subset from APC ChIP-seq is highly enriched for TCF7L2 and AP-1 binding sites

AP-1 (TGASTCA [21]) and TCF7L2 (WWCAAAG [22]) binding motifs occur in 22.6% and 27.0% of all peaks, respectively. Those for SP1 (KGGGCGGRRY [23], 16.1%), USF1/2 (CACGTG [24], 15.0%), NRF1 (GCGCRYGCGC [25], 4.0%) and EGR-1 (GCGKGGGCG [26], 3.6%) occur less frequently. Occurrences of each binding site were then counted within a subset of 500 APC-associated genomic sequences with the highest *p*-values for ChIP-seq enrichment (Figure 3A, columns 6 and 7). All six candidate transcription factor binding motifs show a trend of increasing enrichment as lower-confidence peaks are filtered out, with TCF7L2 (61.8%) and AP-1 (46.8%) binding sites emerging as the best represented and most likely to play roles in APC recruitment.

### APC silencing modifies transcriptional activity driven by TCF7L2 motifs

Luciferase reporter assays were then used to test the hypothesis that APC participates in transcription factor complexes organized around binding sites both for TCF7L2 and for other transcription factors, including NRF1, SP1, EGR-1, USF1/USF2 and AP-1. Three consecutive repeats of NRF1, SP1, EGR-1 USF1/2 or AP-1 consensus binding motifs were cloned into the *pGL3*-promoter reporter vector upstream of the firefly luciferase gene, similar to positive (*TOPFLASH*) and negative control (*FOPFLASH*) reporter vectors containing six repeats of either wild-type or mutant TCF7L2 (formerly known as TCF4) binding sites [3]. *APC* knockdown enhances the ability of the *TOPFLASH* (TCF7L2) positive control to drive luciferase expression by two-fold, while other transcription factor binding motifs show little or no sensitivity to the presence or absence of APC (Supplementary Figure 1). These results support a model in which TCF7L2 is required for gene activation, without excluding the possibility that other transcription factor binding motifs mediate transcriptional sensitivity to APC in the genomic context of a complete regulatory element.

### APC ChIP data show significant overlap with published ChIP data for β-catenin, TCF7L2, AP-1 and SP1

Public ChIP-seq datasets generated from HCT-116 cells were accessed for β-catenin and TCF7L2, as well as for the transcription factors JUND and FOSL1 (components of heterodimeric AP-1 complexes), USF1, SP1 and EGR-1. Data were available from the laboratory of Dr. Richard Myers at the HudsonAlpha Institute for Biotechnology through the NCBI Gene Expression Omnibus [27], and from published work on β-catenin from the laboratories of Dr. Shannon McWeeney and Dr. Gregory Yochum [22]. Chromosomal locations of 3,985 APC-associated genomic regions were compared to 12,375 TCF7L2 peaks, 2,166 β-catenin peaks, 6,969 “AP-1” peaks (shared between JUND and FOSL1), 7,754 SP1 peaks, 3,452 USF1 peaks and 3,396 EGR-1 peaks (Figure 3B) to detect overlap. Fold over-representation was calculated for the occurrence of transcription factor binding site overlap with APC-associated genomic peaks relative to expected occurrences by random chance, with hypergeometric probability testing to calculate *p*-values (Figure 3C). APC peaks overlapped most significantly with TCF7L2 peaks, as well as with SP1, AP-1 and β-catenin peaks. USF1 binding sites exhibited more modest overlap with APC; overlap with EGR-1 binding was not statistically significant (Figure 3C).

### Overlap of APC ChIP-seq with transcription factor binding is more pronounced near genes associated with increased expression following APC loss

**Figure 4:**
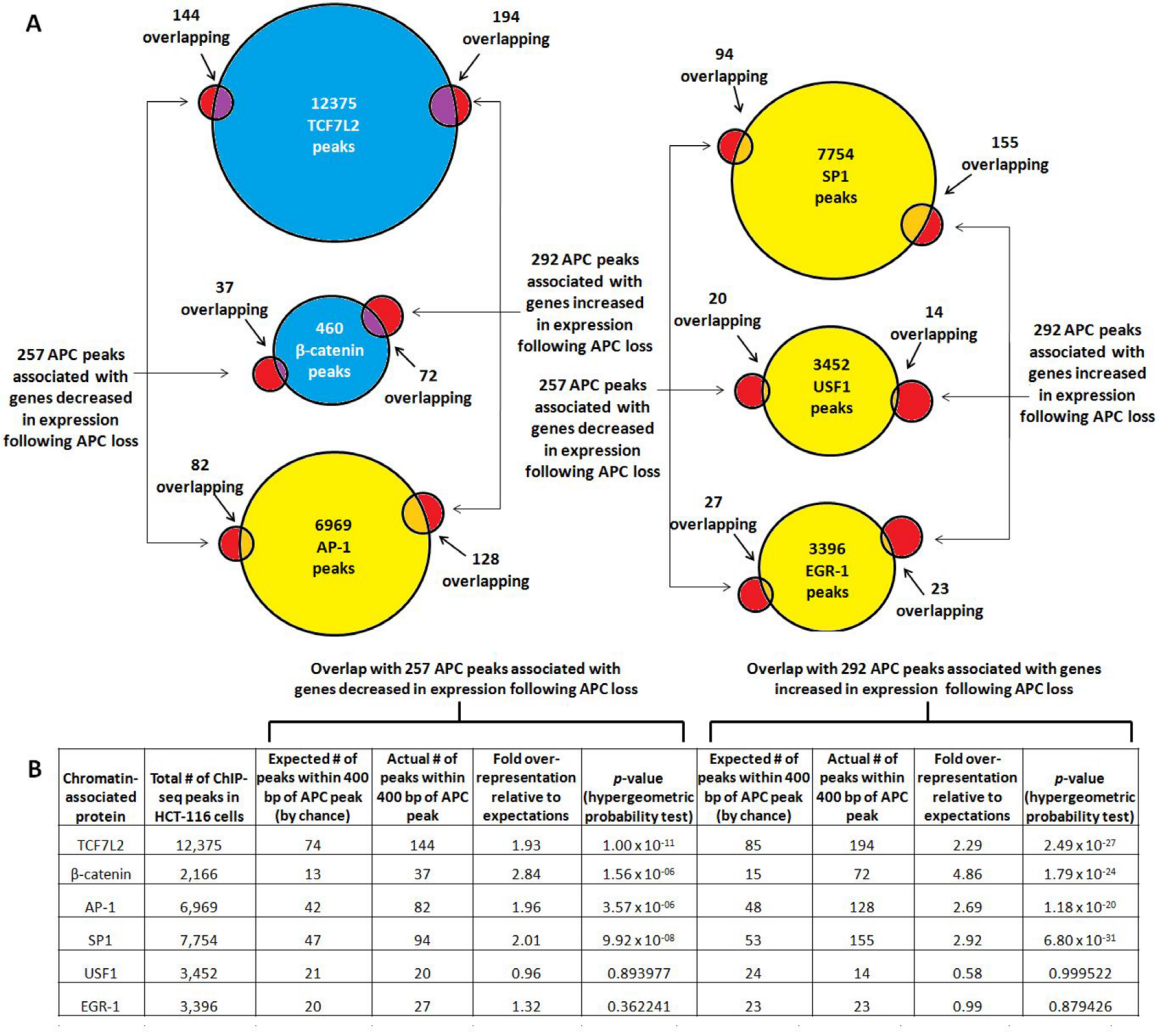
TCF7L2, β-catenin, AP-1 and SP1 bind genomic regions associated with increased expression following APC loss (**A**) Occurrence of validated transcription factor binding sites was examined in 549 genomic sequences enriched by APC ChIP-seq and associated with genes increased (292, red circles on right) or decreased (257, red circles on left) in expression following *APC* silencing. (**B**) Overlap data were tabulated to calculate fold overrepresentation of each transcription factor binding site relative to expected background. The hypergeometric probability test indicated higher significance of overlap for all transcription factors with the subset of APC-associated genomic regions linked to increased as opposed to decreased expression following APC loss (column 10 compared to column 6).

Overlap was re-assessed in the subset of APC-associated genomic regions linked to 257 genes decreased and 292 genes increased in expression following APC loss in HCT-116 cells (Figure 4A). TCF7L2 binding sites showed the most significant overlap with the APC-associated regions, while β-catenin-associated sites occurred with more dramatic over-representation but with less significant *p*-values due to the smaller dataset (Figure 4B). USF1 and EGR-1 binding sites do not occur at higher-than-expected rates in these APC-associated regions. Surprisingly, both AP-1 and SP1 binding sites were overrepresented approximately 2- or 3-fold, and occurred more frequently and with greater significance in regions associated with increased as opposed to decreased expression following APC loss (Figure 4B). These observations were consistent with the role of chromatin-associated APC as a WNT antagonist, as β-catenin and TCF7L2 transcriptionally activate most targets while repressing transcription of a smaller subset [28–33].

### TCF7L2 and AP-1 transcription factor binding sites frequently occur within the same or neighboring genomic regions

Co-occurrence of TCF7L2 and AP-1 binding sites was examined in order to test the hypothesis that these two transcription factor binding sites might coordinately regulate shared target genes. Frequencies were examined in a subset of 549 genomic sequences chosen because of their enrichment by APC ChIP-seq and their proximity to 280 genes altered in expression following *APC* silencing in HCT-116 cells (Supplementary Figure 2). Neighboring genomic regions (defined as those assigned to the same gene locus based on proximity to the nearest transcription start site) were grouped together, making the overlap between TCF7L2 and AP-1 binding sites much more striking (Supplementary Figure 2). TCF7L2 and AP-1 binding sites frequently occur within the same genomic regions, particularly in genomic peaks associated with genes transcriptionally activated following *APC* silencing (Supplementary Figure 2A *vs*. 2B). A similar co-occurrence was observed between TCF7L2 and SP1 binding sites (Supplementary Figure 2C *vs*. 2D). Genomic sequences associated with genes transcriptionally repressed following APC loss contained fewer binding sites for TCF7L2, AP-1 and SP1. These data indicate that TCF7L2 and AP-1 or SP1 transcription factors might bind neighboring genomic regions and converge on the same gene promoter to coordinate transcriptional regulation.

### Target selection and validation

**Figure 5:**
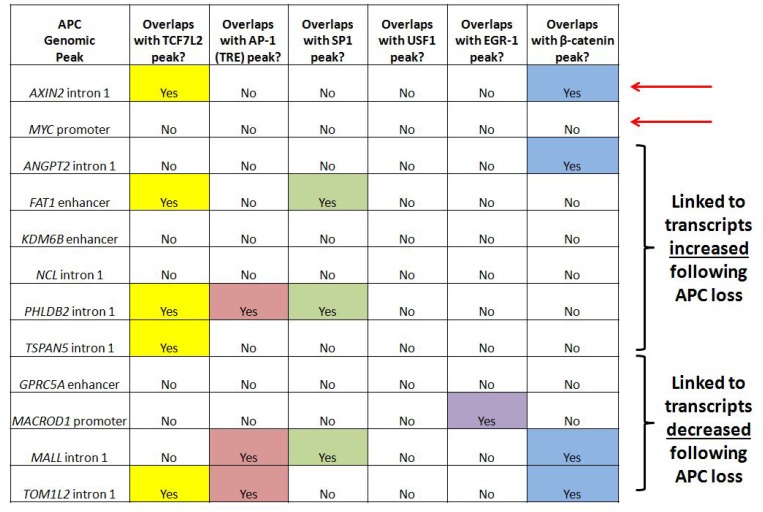
APC-associated candidate genomic regions contain predicted binding motifs for TCF7L2 and AP-1 Transcription factor binding sites identified by ChIP-seq are listed for ten candidate genomic sequences enriched by APC ChIP-seq and associated with transcripts altered in expression following APC loss in both *in vitro* and *in vivo* models. Since many candidate genes are associated with multiple peaks, one peak was chosen in each case based on lowest *p*-value and shortest distance to transcription start site. Most of these genomic sequences contain TCF7L2 and/or AP-1 transcription factor binding sites. Red arrows indicate loci located in *AXIN2* intron 1 and the *MYC* promoter known to be transcriptionally regulated by chromatin-associated APC.

Ten genomic regions of interest were selected for further study (Figure 5) based on differential expression of their encoded transcripts following APC loss in both cell culture (Figure 2B) and mouse colon adenoma (Figure 2C) models, and successful APC ChIP-qPCR validation comparable to positive control peaks clearly visible by qPCR in Supplementary Figure 3B (although the overall purpose of the figure is different). APC-associated regions from the *AXIN2* and *MYC* loci served as positive controls. Analysis of transcription factor ChIP-seq data reveals overlap of four candidate regions with TCF7L2 and three candidate regions with β-catenin binding sites, so that canonical WNT signaling is clearly linked to *ANGPT2* intron 1, the *FAT1* enhancer, *PHLDB2* intron 1, *TSPAN5* intron 1, *MALL* intron 1 and *TOM1L2* intron 1. AP-1 binding sites overlap with the *PHLDB2* intron 1, *MALL* intron 1 and *TOM1L2* intron 1 regions, while SP1 binding sites overlap with the *FAT1* enhancer, *PHLDB2* intron 1 and *MALL* intron 1 regions (Figure 5). The *GPRC5A* enhancer contained predicted TCF7L2 binding motifs not validated by TCF7L2 ChIP (as did the *MYC* promoter, *ANGPT2* intron 1, and *MALL* intron 1, Supplementary Figure 4). Five of these loci contain other APC-associated ChIP-seq peaks collectively characterized by the co-occurrence of TCF7L2 and AP-1 binding sites (Supplementary Figure 4, columns 6, 7, and 8). Regions from the *KDM6B* enhancer, *MACROD1* promoter and *NCL* intron 1 were of interest due to the absence of TCF7L2 or β-catenin binding sites. Their inclusion in subsequent experiments tested the hypothesis that chromatin-associated APC may modulate the transcription of a subset of genes independently of β-catenin or canonical WNT signaling.

### Silencing of ß-catenin expression reduces enrichment of some candidate genomic regions by APC ChIP

ChIP-qPCR experiments were designed to test whether APC requires interaction with β-catenin to associate with chromatin at candidate genomic sequences. ChIP was performed from HCT-116 cells transfected with either scrambled *siRNA* or *siRNA* silencing *CTNNB1* (encoding β-catenin). ChIP antibodies targeted either β-catenin itself (Supplementary Figure 3A) or APC (Supplementary Figure 3B). Reduced enrichment by β-catenin ChIP following transfection with anti-*CTNNB1 siRNA* (Supplementary Figure 3A) was exhibited by both positive controls (from *AXIN2* intron 1 and the *MYC* promoter) and six out of ten candidate sequences (from the *FAT1* enhancer, *PHLDB2* intron 1, *TSPAN5* intron 1, the *GPRC5A* enhancer, *MALL* intron 1 and *TOM1L2* intron 1). Silencing of *CTNNB1* produced more subtle effects on APC ChIP enrichment of target sequences (Supplementary Figure 3B), as only one of the two positive controls (a characterized canonical WNT binding site from *AXIN2* intron 1, but not a site from the *MYC* promoter) and two of the candidate genomic sequences (from *TSPAN5* intron 1 and the *GPRC5A* enhancer) exhibited loss of enrichment. Collectively, these knockdown data indicate that *TSPAN5*, *GPRC5A*, *FAT1*, *PHLDB2*, *MALL* and *TOM1L2* are shared targets of APC and β-catenin, consistent with the widely accepted model that interaction with β-catenin mediates APC recruitment to, and function in, the chromatin fraction [12, 13, 34]. The remaining three candidates (from the *KDM6B* enhancer, *NCL* intron 1 and the *MACROD1* promoter) are inconclusive. Six of the seven putative targets of APC and β-catenin/canonical WNT signaling overlap with TCF7L2 or β-catenin binding sites (Figure 5), while the seventh target, the *GPRC5A* enhancer, has a neighboring peak that exhibits TCF7L2 binding (Supplementary Figure 4, column 7). Overall, the data in Figure 5 and Supplementary Figure 4 indicate that a substantial proportion of APC targets are likely β-catenin- and TCF7L2-dependent.

### Candidate genomic regions were screened by luciferase reporter assay for APC- or β-catenin-dependent changes in their ability to drive transcription

**Figure 6:**
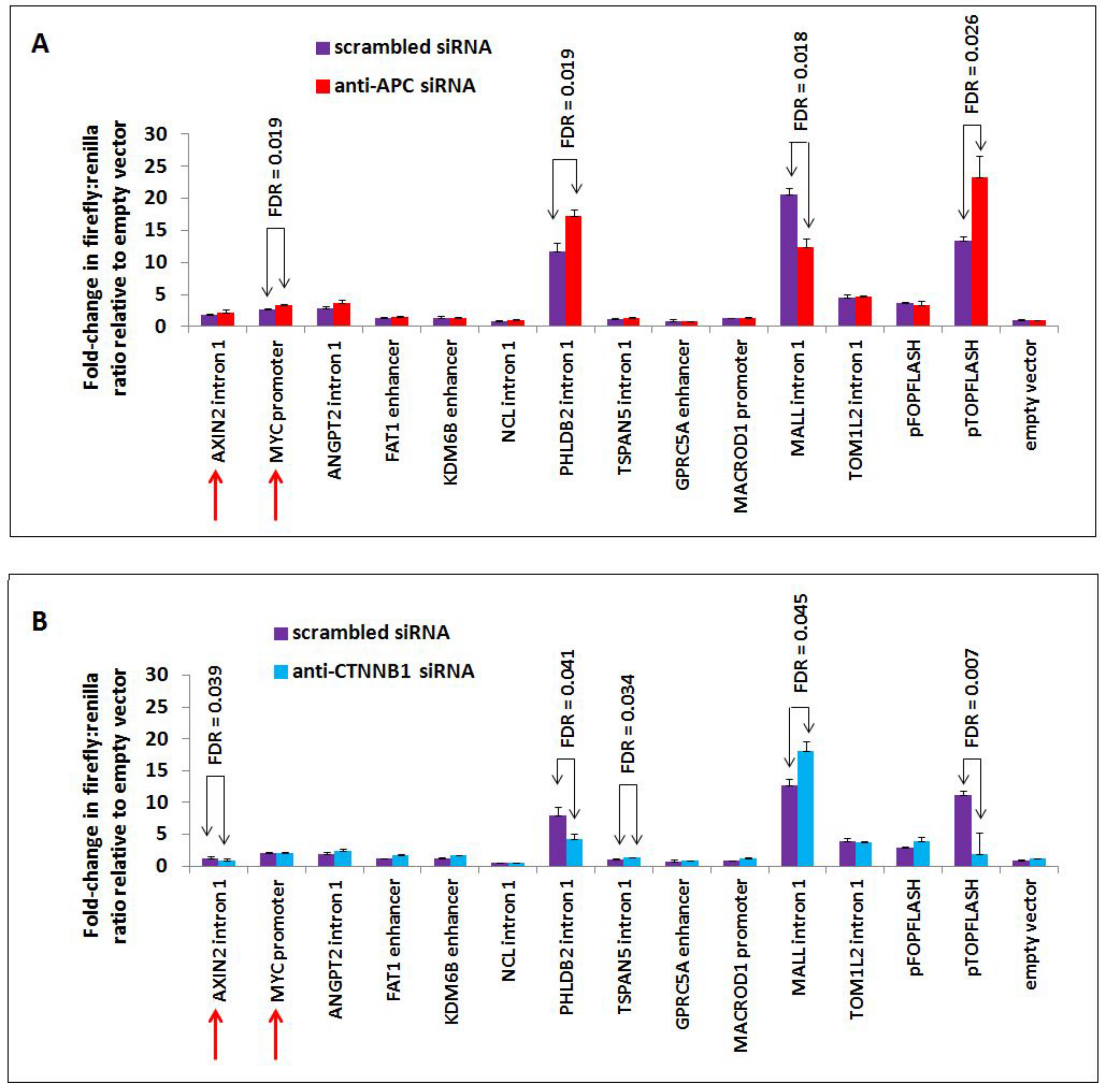
*APC* or *CTNNB1* silencing alters transcriptional activity from elements in the *PHLDB2* and *MALL* loci Ten genomic sequences associated with APC and two positive control sequences (from *AXIN2* intron 1 and *MYC* promoter, indicated by red arrows) were PCR-amplified, cloned into the *pGL3*-promoter firefly luciferase vector and transfected into HCT-116 cells previously transfected with either scrambled *siRNA* (purple) or *siRNA* targeting *APC* (red, panel **A**) or *CTNNB1* (blue, panel **B**). The *PHLDB2* intron 1 construct was more active following *APC* silencing (panel A) and less active following *CTNNB1* silencing (panel B), similar to the *pTOPFLASH* positive control. The *MALL* intron 1 construct was less active following *APC* silencing and more active following *CTNNB1* silencing. Errors bars are based on standard deviation, and FDR values lower than 0.05 are indicated (1-tailed Student's *t*-test).

Previous studies have constructed a model in which APC antagonizes canonical WNT activation of target genes [12, 13]. Interestingly, we find that not all β-catenin-dependent target genes of chromatin-associated APC increase in expression following *APC* silencing or mutation, as *GPRC5A*, *MALL* and *TOM1L2* decrease following APC loss *in vitro* (Figure 2B) and *in vivo* (Figure 2C). These data demonstrate that APC associates with chromatin at WNT-repressed target genes as well, likely via a similar mechanism of interaction with β-catenin. This was further tested using luciferase reporter assays to characterize the genomic sequences enriched by APC ChIP-seq (Figure 6). Peaks of interest (approximately 0.5–1.0 kb) were PCR-amplified, cloned into the *pGL3*-promoter firefly luciferase vector and transfected into HCT-116 cells to measure their ability to drive luciferase expression either in the presence or absence of *siRNA* to *APC* (Figure 6A) or *CTNNB1* (Figure 6B).

**Figure 7:**
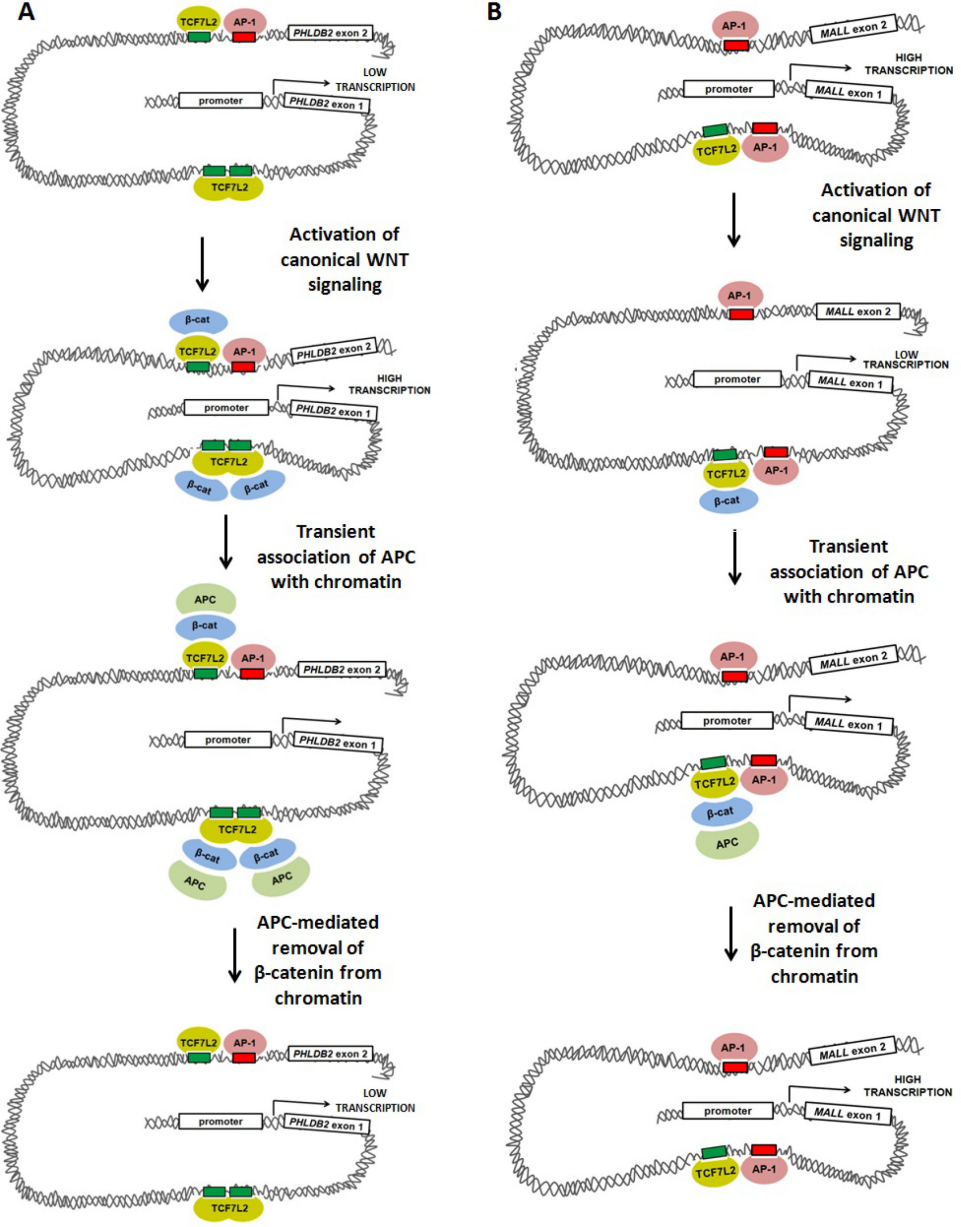
Models of WNT-mediated transcriptional activation and repression involving TCF7L2, AP-1, β-catenin and APC Our data suggest at least two models in which canonical WNT-mediated transcriptional activity, APC and AP-1 alter gene expression. (**A**) Activation of canonical WNT signaling up-regulates transcription of *PHLDB2* through a previously-characterized mechanism in which β-catenin binds to the TCF7L2 transcription factor, promoting DNA bending that brings transcriptional machinery (including AP-1) into closer association with the proximal promoter. APC disrupts this association by mediating the removal of β-catenin from the complex. (**B**) Activation of canonical WNT signaling may down-regulate AP-1-dependent transcription of *MALL* through a mechanism in which β-catenin binding to TCF7L2 disrupts interaction of transcriptional machinery with the proximal promoter. APC may relieve this disruption by removing β-catenin from the complex.

Many of the candidate luciferase constructs were not sufficient to drive transcription of the reporter above baseline levels, including the *AXIN2* intron 1 and *MYC* promoter positive controls. Other candidates showed only limited responsiveness to *siRNA* reducing the expression of APC. Candidate sequences from *PHLDB2* intron 1 and *MALL* intron 1 exhibited high transcriptional activity and strong responses to both *APC* and *CTNNB1* silencing. *APC* silencing by *siRNA* transfection increased luciferase activity from the 541-bp *PHLDB2* intron 1 construct (Figure 6A), while silencing of *CTNNB1* (encoding β-catenin) decreased luciferase activity (Figure 6B). These results indicate that this genomic region contributes to the activation of *PHLDB2* transcription following *APC* loss both observed both *in vitro* (Figure 2B) and *in vivo* (Figure 2C). The reporter construct containing a region of *MALL* intron 1 decreased its ability to drive transcription following *APC* silencing (Figure 6A) and increased its activity following *CTNNB1* silencing (Figure 6B). Both observations indicate that this genomic region contributes to the transcriptional activation of *MALL* following *APC* loss (Figure 2B and 2C). It is important to note that this effect likely is mediated not only by the loss of APC function as a direct regulator of β-catenin in the partially chromatinized context of the luciferase construct, but also by the up-regulation of β-catenin protein levels that typically follows the loss of cytoplasmic APC as a negative regulator of β-catenin stability. Expression profiling data confirming that *Phldb2* increases in expression while *Mall* decreases in expression in mouse adenomas relative to adjacent non-adenoma tissue (Figure 2C) match reports that canonical WNT signaling activates the transcription of certain genes and simultaneously represses the transcription of others [28–33]. Collectively, the results of this study indicate that chromatin-associated APC may function to reverse the effects of canonical WNT signaling on both activation and repression of targets (Figure 7).

## DISCUSSION

This study has identified high-confidence APC targets genes by comparing mechanistic (ChIP-seq) and functional (RNA-seq) data, similar to other studies identifying WNT-activated target genes [35, 36]. The present study adds evidence that canonical WNT signaling represses distinct target genes in human colorectal cancers and that APC antagonizes WNT repression at these loci. This pattern differs from the better-characterized canonical WNT targets but matches a smaller group of targets found in model systems such as *Drosophila* [28, 29], chick [30], mouse [31, 32] and human melanocytes [33].

The mechanism by which this occurs remains unclear. Published studies of chromatin-associated APC have established its ability to antagonize canonical WNT transactivation of targets, and have shown that interaction with β-catenin and recruitment of co-repressors such as CtBP-1, TLE-1 and HDAC1 are key elements of that mechanism [12, 13, 34]. APC and CtBP-1 interact with these loci transiently, and their disappearance coincides with the loss of β-catenin and the appearance of the more stable co-repressors [12]. Our findings indicate that the antagonistic relationship between chromatin-associated APC and β-catenin exists at WNT-repressed genes such as *MALL* as well. We hypothesize that regardless of whether canonical WNT signaling activates or represses transcription of a particular gene, APC exerts an antagonistic effect by mediating β-catenin removal (Figure 7). Further mechanistic experiments will be necessary to test this hypothesis and to identify co-activators involved in APC regulation of genes such as *MALL*.

Among the candidate genes identified in this study are seven characterized β-catenin-dependent targets of chromatin-associated APC. The *ANGPT2*, *FAT1*, *PHLDB2* and *TSPAN5* transcripts increase while *GPRC5A*, *MALL* and *TOM1L2* decrease following *APC* loss *in vitro* or *in vivo*. An intronic regulatory element from the *PHLDB2* locus is transcriptionally activated by β-catenin/TCF7L2 and repressed by APC, while an intronic regulatory element from the *MALL* locus is transcriptionally repressed by β-catenin/TCF7L2 and activated by APC. It is important to note that the effects of APC on their transcription are likely mediated by both cytoplasmic and nuclear functions of the APC protein that cannot be distinguished in our reporter assays. Previously published studies of the relationship between chromatin-associated APC and canonical WNT signaling indicate an antagonistic relationship that is consistent with the functions of APC in facilitating nuclear export and cytoplasmic degradation of β-catenin. The co-existence of these three seemingly redundant mechanisms emphasizes the critical importance of APC as a switch whose activation in maturing cells of the colorectal epithelium triggers rapid and tight negative regulation over the nuclear functions of β-catenin at multiple levels.

The chromatin-associated fraction of the APC tumor suppressor protein associates with a large number and variety of genomic regions, with patterns in genomic distribution and transcription factor binding site content that match ChIP-seq studies of other known components of the canonical WNT signaling pathway [22, 35]. Binding sites for the TCF7L2 transcription factor are enriched among APC-associated regions (Figures 3, 4), similar to published ChIP-seq results for β-catenin [22], a known co-regulator of TCF7L2 transcription factor complexes. These similarities are consistent with the reported co-localization of both proteins to target sites in the chromatin fraction and the interpretation that APC can be recruited to chromatin by β-catenin binding [12]. Despite similarities to related studies, it is important to note the limitations of informatics analyses that are two-dimensional and assign ChIP-seq peaks to RNA-seq hits based on the assumption that each APC-bound region controls gene expression from the nearest transcription start site, where the more realistic spatial organization of chromatin in three dimensions enables regulatory relationships that are not as long-range as they appear in two-dimensional units (bp). It is particularly important to consider these limitations in light of the heterogeneous effects of APC on “control” loci such as *SP5*, which lacks obvious APC-associated sites according to ChIP-seq, but nevertheless shows sensitivity to *APC* loss in the RNA-seq data. Three-dimensional analyses based on chromatin conformation capture (3C) methodologies are required for a more comprehensive identification of the genes targeted by APC-associated regions genome-wide.

Mounting evidence links β-catenin [22, 37, 38] and now APC to genomic regions containing predicted AP-1 binding sites. Canonical WNT signaling shares transcriptional targets with the AP-1 signaling pathway [37, 38], and previous ChIP-seq targeting β-catenin [22] similarly found AP-1 binding motif enrichment. The AP-1 component c-JUN physically interacts with TCF7L2 in HCT-116 cells, co-regulating the *JUN* promoter in a β-catenin-dependent manner [37]. AP-1 components co-immunprecipitate with β-catenin and co-regulate expression of the TCF7L2 target genes *MYC* and *CCND1* [38]. Co-occurrence of predicted TCF7L2 and AP-1 binding sites in many of the same or neighboring genomic regions (Supplementary Figure 2) indicates that the two transcription factors may coordinately regulate the transcription of shared target genes in the colorectal epithelium. SP1 transcription factor binding sites are similarly over-represented among APC ChIP-seq peaks (Figures 3, 4) and co-occur with TCF7L2 binding sites (Supplementary Figure 2).

It is as yet unclear what determines whether canonical WNT signaling activates or represses a particular target gene in human cells. Some correlation exists between the presence of TCF7L2 and AP-1 transcription factor binding sites and canonical WNT activation of transcription, as both types of binding site are more prevalent within the subset of genomic sequences associated with transcripts increased in expression following *APC* loss (Supplementary Figure 2A vs. 2B). However, all seven loci characterized in this study include APC-associated genomic regions that contain both TCF7L2 and AP-1 binding motifs, regardless of whether they are transcriptionally increased (*ANGPT2*, *FAT1*, *PHLDB2* and *TSPAN5*) or decreased (*GPRC5A*, *MALL* and *TOM1L2*) following *APC* silencing.

β-catenin/TCF7L2 may control transcription by modulating AP-1 binding or subsequent AP-1-dependent steps. If canonical WNT signaling exerts transcriptional effects largely by modifying AP-1 activity on shared target genes, WNT-activated targets may be those in which TCF7L2 promotes AP-1 binding or coordinates long-distance interactions between AP-1 proteins from multiple binding sites at the same promoter (Figure 7A). This would be consistent with the observation that many target genes (including *PHLDB2*) have multiple associated peaks, some with either TCF7L2 or AP-1 binding sites, and some with both. These regulatory elements likely interact with one another and converge on the promoter to co-regulate transcription. According to this model, WNT-repressed targets (Figure 7B) might be those in which TCF7L2 instead competes with AP-1 binding to adjacent sites or interferes with long-distance interactions between AP-1 proteins and their intended target promoters.

The *MALL* gene was confirmed (Figure 6) as a direct APC target repressed by canonical WNT signaling. These data imply that other targets with similar expression patterns may be similarly regulated candidates. Their value as potential markers for prognosis or as therapeutic targets may be important. *MALL* is down-regulated in colorectal cancers [39], with this low expression predicting tumor recurrence, metastasis and poor outcome [40]. MALL is a member of the glycolipid-enriched membrane raft family [41] and interacts with caveolin-1 [42], potentially linking it to integrin signaling and cell migration [43]. High expression of *PHLDB2* in colorectal cancer is also associated with shorter metastasis-free survival. PHLDB2 is required for cell migration and invasion of the HCT-116 colon cancer cell line [44]. It is regulated by PI3K [45] and contributes to microtubule stabilization [46], and helps promote cell protrusions by linking actin and adhesion dynamics [47]. *MALL*, *PHLDB2* and other genes responsive to APC are consistent with the emerging role of canonical WNT signaling targets in colorectal cancer invasion and progression [48–50], in addition to the better-characterized role of the pathway in colorectal tumor initiation.

Finally, this study strongly suggests that targets of the canonical WNT signaling pathway should be stratified into several categories, based on criteria such as the direction of their transcriptional response to activation of the pathway. The high rates of co-occurrence of TCF7L2 and AP-1 binding sites observed in the ChIP-seq data further indicate that the presence or absence of AP-1 binding sites may be an additional criterion by which to sort target genes. Interestingly, the AP-1 component c-Jun is required for the full phenotype of *Apc* loss in mice, as introduction of *Jun* mutations into the *Apc^Min/+^* mouse model lead to lower polyp number, reduced polyp size and longer life span [37]. Canonical WNT signaling promotes the maintenance of progenitor cell phenotypes in the stem cell compartment of the colorectal epithelium, and may do so in part by fine-tuning the transcription of target genes shared with AP-1. Modulation of the AP-1 pathway is increasingly feasible with the emergence of the small molecule T-5224, designed to inhibit transactivation by blocking DNA binding by the leucine zipper domain of c-FOS [51, 52]. T-5224 can be one interventional strategy for WNT-driven colorectal tumors and has been characterized as an agent for the treatment of inflammatory conditions such as rheumatoid arthritis [52] and injuries of the kidneys [53] and liver [54] as well as oral cancer [55]. The natural product veratramine is an additional small molecule inhibitor of AP-1 that inhibits transactivation by interacting not with AP-1 itself but with its TGACTCA binding motifs [56]. These agents may show potential as therapeutic interventions to reduce the contribution of shared TCF7L2/AP-1 target genes to colorectal tumor progression.

## MATERIALS AND METHODS

### Chromatin immunoprecipitation and next-generation sequencing

APC ChIP was performed from 60–70% confluent HCT-116 cells in a 150-mm dish. Cells were maintained in McCoy's 5A medium including glutamine and supplemented with 10% FBS. Following formaldehyde crosslinking, nuclei were isolated and resuspended in 1 mL of Lysis Buffer (25 mM Tris-HCl pH 7.5, 150 mM NaCl, 5 mM EDTA, 0.1% SDS, 1% Triton X-100, 0.5% Sodium Deoxycholate, 1 mM PMSF with mammalian protease inhibitor cocktail). Probe-based sonication was performed at 4° C over a period of 40 minutes total by 30 pulses of 10-seconds each at 35% amplitude. Average fragment size was further reduced to ~1kb using micrococcal nuclease (Affymetrix, Inc.). Input material was pre-cleared by 2-hour incubation with 5 μg rabbit IgG antibody and 40 μL of pre-equilibrated Protein G Dynabead slurry (Thermo Fisher Scientific) at 4° C with rotation. 100 μL of the resulting supernatant was saved as “pre-cleared input”, and the remainder was used for α-APC ChIP in combination with 10 μg of α-APC antibody (a polyclonal antibody recognizing the C-terminal 50 amino acids of APC, catalog #A3081A, Bethyl Laboratories, Inc.). ChIP was performed according to an established protocol [57], with the following exceptions. 40 μL of pre-equilibrated Protein G Dynabead slurry (Thermo Fisher Scientific) was used to pull down antibody-APC complexes, and each of the wash steps was performed twice. Yields from multiple parallel α-APC ChIP reactions were pooled to obtain the 10 ng required for ChIP-seq library preparation, and the PCR Purification Kit (QIAGEN) was used to clean up reactions prior to library preparation. Two separate biological replicates were performed under identical conditions several months apart, from HCT-116 cells at passage numbers between 10 and 20 since they were purchased from the American Type Culture Collection. Library preparation was performed using the NEBNext ChIP-Seq Library Prep Master Mix Set for Illumina, using adaptors AD005 and AD019 (New England Biolabs) for input and ChIP libraries, respectively. Size selection was performed by E-gel (Life Technologies, Inc.) to obtain fragments of 300-400-bp in size. Next-generation sequencing (50-bp, single-end) was performed by the OSU Genomics Shared Resource using a HiSeq 2500 instrument (Illumina, Inc.).

### ChIP-seq data analysis

97–98% of reads for two ChIP-seq samples and the matching input samples passed quality control filters, and the Burrows-Wheeler Aligner generated bam alignment files using UCSC hg19. The Model-based Analysis of ChIP-Seq version 2 (MACS2) tool [58] performed peak-calling and generated peak scores, *p*-values and false discovery rates (FDR) for each peak. Peak score threshold was adjusted to lower the noise observed in input files while retaining internal positive control peaks in the *AXIN2*, *DKK1* and *MYC* loci. The RefGene database was used to annotate peak regions, determine distances to transcription start sites and assign peaks to genes. The distribution of peak locations relative to transcription start sites is shown in Supplementary Figure 5. R software identified overlapping peaks present in both replicates with summits separated by less than 400-bp (the median of peak width in the peak calling results). ChIP-seq datasets are available through NCBI's Gene Expression Omnibus (accession #GSE99264, https://www.ncbi.nlm.nih.gov/geo/query/acc.cgi?acc=GSE99264). Reviewers must use the secure token uzojsoqgddktvev. *MEME-ChIP* [19], *Regulatory Sequence Analysis Tools* (*RSAT*) [20], and *MatInspector* [18] algorithms were used with standard/default settings to perform initial analyses of all ChIP-seq peak sequences from FASTA files.

### Whole transcriptome profiling of HCT-116 cells

HCT-116 cells were transfected on consecutive days with pooled *siRNA* (Dharmacon) targeting *APC* (L-003869-00-0005), scrambled sequence (D-001810-10-05), or neither (mock transfection). Cells were harvested 48 hours later by standard Trizol (Life Technologies catalog # 15596-026) isolation protocol. Single-read library preparation was performed using the TruSeq RNA Library Preparation Kit (Illumina catalog # RS-122-2001). Next-generation sequencing was performed by the OSU Genomics Shared Resource using an Illumina Genome Analyzer II instrument. The Cufflinks software program [14] assigned reads to transcripts, performed quantification and calculated statistical significance. The Cuffdiff program provided transcript levels (as FPKM) and (FDR-adjusted) *q*-values based on fold-changes and sample sizes and identified significantly significant differences between anti-APC *siRNA* and scrambled *siRNA*-transfected conditions (*q* < 0.05). RNA-seq datasets are available through the NCBI Gene Expression Omnibus (accession #GSE99264).

### Whole transcriptome profiling of Apc^Min/+^ and AOM/DSS mouse colon tumors

RNA isolation, preparation of sequencing libraries and processing of these data were described previously [15]. The presence of activating mutations in *Ctnnb1* was confirmed by Sanger sequencing of cDNA from the adenomas from AOM/DSS-treated mice (Supplementary Figure 6). Transcripts of interest were identified based on 1.5-fold-changes in expression in adenomas from both *Apc^Min/+^* and AOM/DSS-treated mice relative to their respective non-adenoma colon tissue controls (using one-sample *t*-test with a significance cutoff of FDR < 0.05) in the same direction as was observed in human cells following *APC* silencing. For multiple test correction, fold-changes in expression for individual genes were ordered by *p*-value and ranked, and FDR was calculated by multiplying *p*-values by the total number of tests and dividing by their rank (Benjamini and Hochberg method). RNA-seq datasets are available through NCBI's Gene Expression Omnibus (accession #GSE98496, https://www.ncbi.nlm.nih.gov/geo/query/acc.cgi?token=wpidaoiuphunvcn&acc=GSE98496).

### APC ChIP-seq overlap with publicly available transcription factor ChIP-seq datasets

Public ChIP-seq datasets were obtained through accession numbers GSM1010846 (EGR-1) GSM1010756 (FOSL1), GSM1010847 (JUND), GSM803474 (POLR2A), GSM1010902 (SP1), GSM782123 (TCF7L2), and GSM1010836 (USF1). The coordinates of β-catenin ChIP-seq peaks were obtained from published work from the laboratories of Dr. Shannon McWeeney and Dr. Gregory Yochum [22]. All datasets included lists of peaks from two ChIP-seq replicates (except for β-catenin); peaks for each transcription factor were narrowed down to only those shared between both replicates. Chromosomal locations of 3,985 APC-associated genomic regions were compared to 12,375 TCF7L2 peaks, 2,166 β-catenin peaks, 6,969 “AP-1” peaks (shared between both JUND and both FOSL1 replicates), 7,754 SP1 peaks, 3,452 USF1 peaks and 3,396 EGR-1 peaks. Overlap between an APC peak and a transcription factor peak was scored only when the centers of two corresponding peaks were no more than 400-bp apart.

### Luciferase reporter assays

Genomic regions were PCR amplified, restriction digested and ligated into the *pGL3*-promoter vector upstream of the firefly luciferase gene. Restriction enzymes pairs varied depending on the target, with *Kpn I* and *Bgl II* used for the majority. Primer sets are listed in Supplementary Figure 7. HCT-116 cells were seeded in 6-well plates and transfected on consecutive days with pooled *siRNA* (Dharmacon) targeting *APC* (L-003869-00-0005), targeting *CTNNB1* (encoding β-catenin, L-003482-00-0010), or with scrambled sequence (D-001810-10-05) using Dharmafect 2 reagent (T-2002-01). 12 hours later, cells were trypsinized and re-seeded into 96-well plates at a density of 25,000 cells per well. Cells were co-transfected 12-hours post-plating with a firefly luciferase construct (9 ng per well) and a Renilla luciferase construct (1 ng per well). Luciferase assays were performed 24 hours later using the Promega Dual-Luciferase Reporter Assay System (catalog #E1910). The ratio of firefly luciferase to Renilla luciferase signal for each well was normalized to the average across all wells transfected with empty vector. Three independent experiments were performed and two-tailed Student's *t*-test was used to calculate *p*-values to test the hypothesis that each construct was APC-sensitive or β-catenin-sensitive, relative to its scrambled *siRNA* control. For multiple test correction, fold-changes in expression for individual constructs were ordered by *p*-value and ranked, and FDR was calculated by multiplying *p*-values by the total number of tests and dividing by their rank (Benjamini and Hochberg method).

### Ethics statement

The OSU Institutional Animal Care and Use Committee (IACUC) provided prior approval for all experiments involving mouse tissues (OLAW Assurance # A3261-01). IACUC approved the animal use protocol #2012A00000021, and animal work was conducted in accordance with its established criteria. Decisions to remove mice from the animal facility were made in conjunction with veterinary staff, and mice were observed and evaluated daily for predetermined criteria necessitating removal and euthanasia. Carbon dioxide inhalation followed by cervical dislocation was used to euthanize mice.

## SUPPLEMENTARY MATERIALS FIGURES


