# Supplementary material for: Chromatin-associated APC regulates gene expression in collaboration with canonical WNT signaling and AP-1
Source: Oncotarget. 2018 Jul 27;9(58):31214–30. doi: 10.18632/oncotarget.25781 (PMC6101278; doi:10.18632/oncotarget.25781)
Supplement: Supplementary file 1 [file oncotarget-09-31214-s001.pdf]

Chromatin-associated APC regulates gene expression in collaboration with canonical WNT signaling and AP-1

SUPPLEMENTARY MATERIALS

ChIP-qPCR with silencing of β-catenin expression

Chromatin immunoprecipitation with anti-APC antibody was performed as previously described, while ChIP for β-catenin was similar except for the substitution of 10 µL of rabbit anti-β-catenin antibody (Cell Signaling Technology catalog #8480S). Cleanup of ChIP reactions was performed using the QIAGEN PCR purification kit, and 0.5–2 µL of the 50 µL elution volume was sufficient for qPCR amplification using most primer sets of interest. Approved primer sets had each been previously optimized to confirm that C_T values of 30 cycles or fewer could be obtained within a linear range and with a single observed melting temperature. This volume of template was adjusted to a total volume of 6 µL using nuclease-free water, combined with 4 µL total of diluted qPCR primers (1.5 µM each primer, see Supplementary Figure 9 for sequences), and mixed with 10 µL of 2× Power SYBR Green PCR master mix (Thermo Fisher Scientific catalog # 4367659). For each ChIP reaction, corresponding input material was purified in parallel and analyzed by qPCR using 5–20 ng of purified input DNA as template. qPCR reactions were performed by BIORAD iCycler Real-Time PCR Detection System. qPCR reactions were performed as three technical replicates of each biological sample. Measurements from three independent biological replicates were statistically analyzed by one-tailed Student's *t*-test.

Generation of luciferase constructs by direct ligation

Luciferase reporter constructs for transcription factor binding sites of interest were generated by annealing the complementary primer sets (Figure 7A), ligating them directly (using sticky ends on the antisense primer) into the *Kpn I*-, *Bgl II*-cut *pGL3*-promoter vector, and confirming the desired insertion by Sanger sequencing. Each primer set contains three consecutive repeats of the binding site of interest, separated by 7-14-bp spacing linkers similar to those found within the positive control *pTOPFLASH* construct.

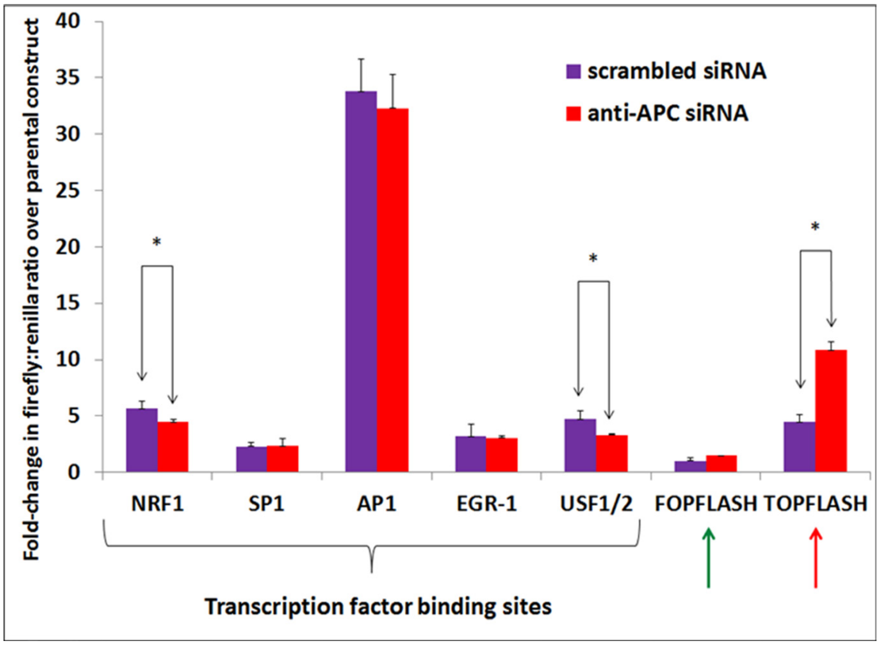

Supplementary Figure 1: Only the TCF7L2 transcription factor binding motif mediates transcriptional changes upon APC silencing *in vitro*. Three consecutive repeats of each predicted transcription factor binding site were cloned into a luciferase reporter vector and co-transfected with a Renilla luciferase plasmid into HCT-116 cells previously transfected either with scrambled *siRNA* (siSCR, in purple) or anti-APC *siRNA* (siAPC, in red). The negative control (*FOPFLASH*, green arrow) and positive control (*TOPFLASH*, red arrow) indicated that the anti-*APC siRNA* successfully upregulated canonical WNT signaling, while the constructs exhibited minimal changes in transcription following *APC* silencing, indicating that most candidate transcription factor binding sites are relatively APC-insensitive outside of their genomic context.

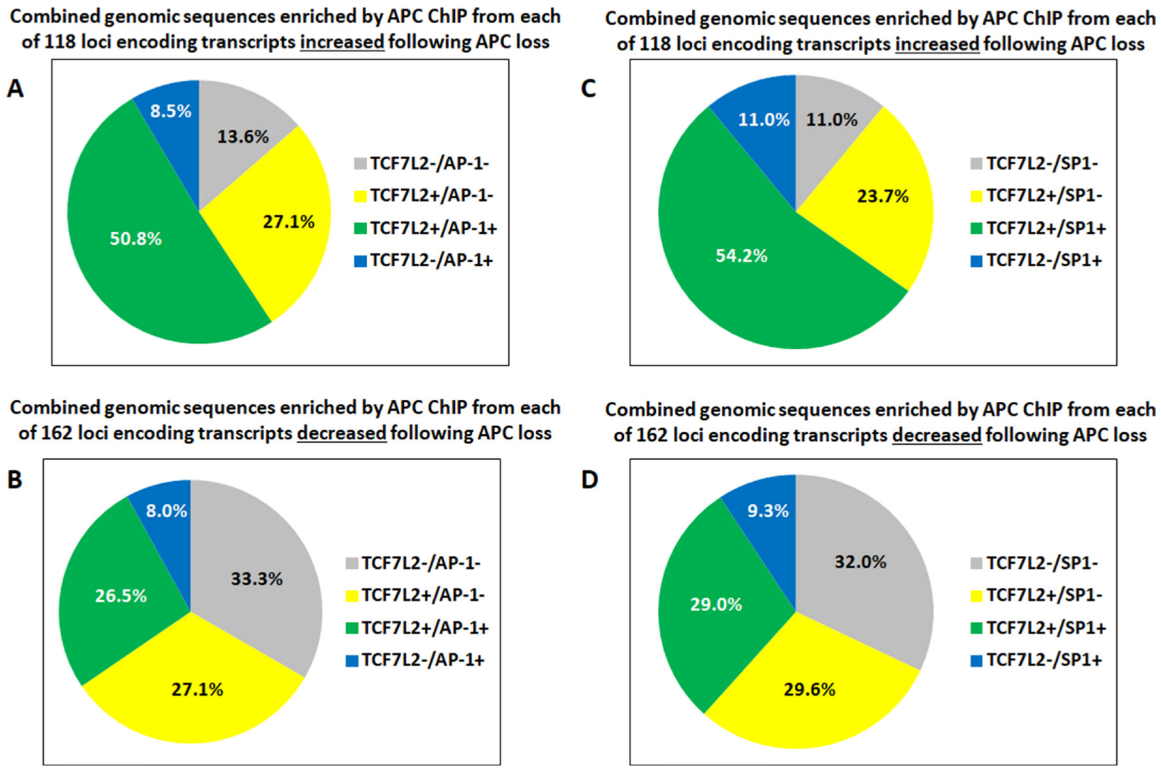

Supplementary Figure 2: TCF7L2 binding sites co-occur with AP-1 and SP1 sites in APC-associated genomic regions. The co-occurrence of TCF7L2 with either AP-1 or SP1 binding sites was examined in 549 genomic sequences enriched by APC ChIP-seq and associated with 280 genes changed in expression following *APC* silencing. For genes associated with multiple APC-associated regions, neighboring genomic regions were grouped together to take into account the co-occurrence of TCF7L2 and AP-1 binding sites in adjacent APC peaks. TCF7L2 and AP-1 binding sites frequently occur within the same genomic regions, particularly in genomic peaks associated with genes increased in transcription following *APC* silencing (**A**) in comparison to (**B**). A similar co-occurrence was observed between TCF7L2 and SP1 binding sites (**C**) in comparison to (**D**). Thus, TCF7L2 binding sites frequently occur in the same or in adjacent genomic sequences with AP-1 and/or SP1 and might coordinately regulate transcription of these shared target genes.

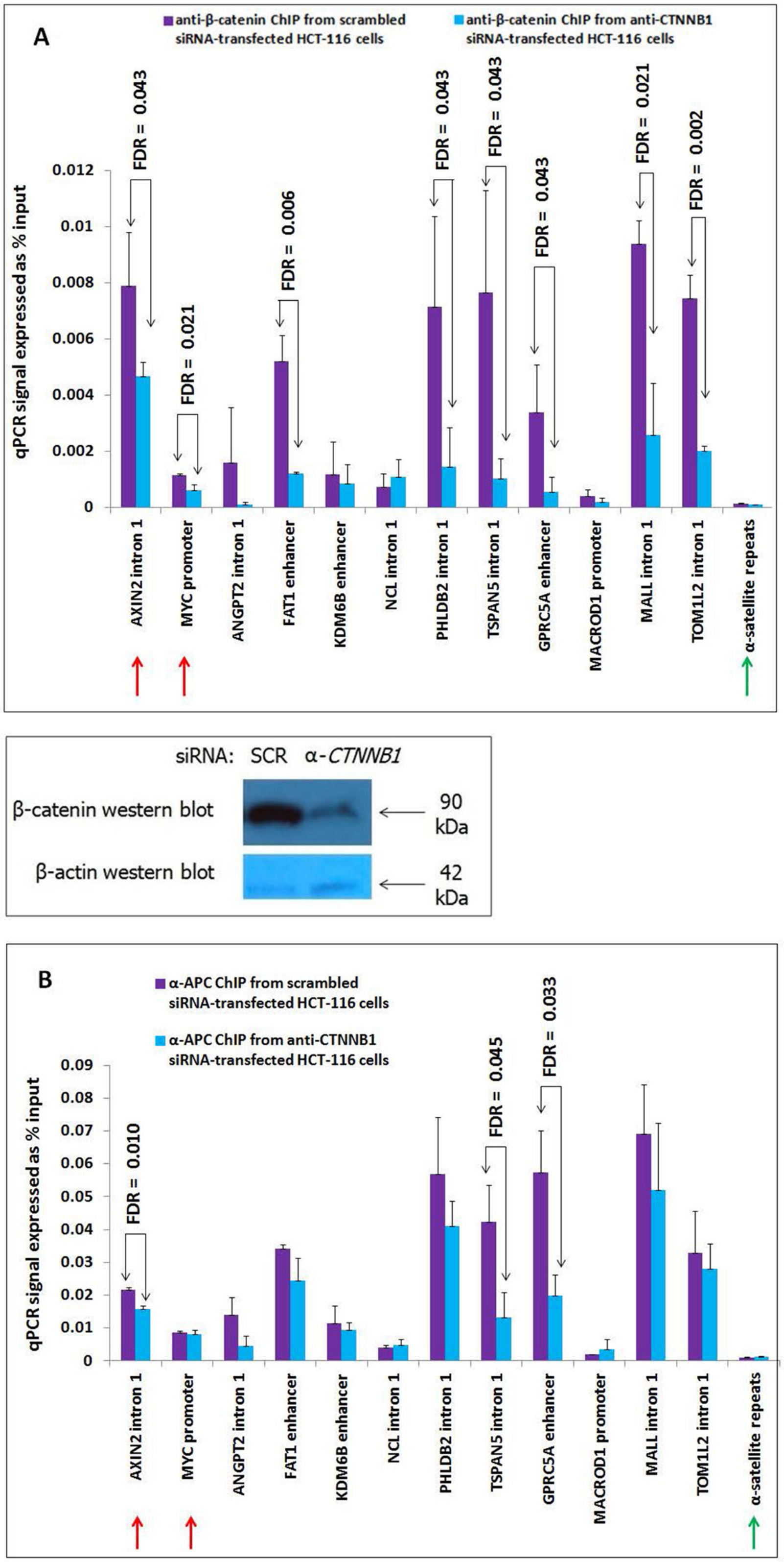

Supplementary Figure 3: *CTNNB1* silencing significantly abolishes β-catenin but not APC ChIP enrichment of most candidate APC targets. Twelve genomic regions enriched by APC ChIP were examined by ChIP-qPCR using anti-β-catenin (**A**) and anti-APC (**B**) antibodies. ChIP was performed from HCT-116 cells transfected with either scrambled siRNA (siSCR, in purple) or siRNA targeting β-catenin (siCTNNB1, in blue). α-satellite repeats (green arrow) were used as a negative control target, while target sequences in *AXIN2* intron 1 and the *MYC* promoter (red arrows) were positive controls regulated by canonical WNT signaling as well as enriched by APC ChIP-seq. Silencing β-catenin significantly reduced β-catenin ChIP-qPCR signal for most genomic targets but reduced APC ChIP-qPCR signal to less significant degree in most cases. Error bars are based on standard deviation and statistical significance was calculated from three biological replicates using the Student's *t*-test (FDR < 0.05).

Candidate Gene	siSCR fold-change	siAPC fold-change	AOM/DSS fold-change	*Apc*$^{Min/+}$ fold-change	Total # of peaks	# TCF7L2^{+} peaks	# AP-1^{+} peaks
ABCC3	-1.08	-3.16	-1.68	-3.74	2	1	2
GALNT7	-0.95	-1.47	-2.73	-4.68	2	2	1
GPRC5A	-1.17	-1.48	-3.94	-3.82	6	4	2
IL18	-1.07	-1.50	-3.85	-3.15	1	1	1
MACROD1	-0.86	-1.85	-2.00	-1.60	1	0	0
MALL	-0.99	-2.01	-5.32	-4.62	4	1	3
NT5E	-0.77	-1.51	-2.28	-1.85	1	0	1
SCNN1A	-1.13	-2.03	-4.11	-5.63	1	0	0
TOM1L2	-1.10	-1.92	-1.72	-1.85	3	1	1
ANGPT2	3.94	5.67	2.07	7.42	2	1	1
ANXA1	1.06	1.44	1.54	2.29	1	0	1
AXIN2	1.08	7.00	9.87	22.85	5	3	1
FAT1	1.06	1.27	1.56	1.65	13	8	7
KDM6B	1.03	1.62	1.75	2.57	2	0	0
MALT1	1.06	1.74	1.77	1.56	1	1	1
MSX1	0.97	3.02	1.61	4.76	4	3	1
NCL	0.98	1.07	1.86	2.30	1	0	0
NR4A2	0.93	2.03	2.38	3.00	2	1	0
PHLDB2	1.02	1.36	2.25	3.25	17	8	4
RHEBL1	1.60	2.16	2.17	1.67	1	0	0
STX16	1.00	1.28	2.76	2.19	1	0	0
TEX14	1.16	1.73	2.91	3.68	3	1	0
TMOD3	1.04	1.42	2.53	3.04	4	1	2
TSPAN5	0.98	1.68	1.53	3.33	4	3	2
ZNRF3	1.06	1.76	3.32	5.19	4	4	0

Supplementary Figure 4: High confidence targets of chromatin-associated APC are associated with TCF7L2 and AP-1 binding sites. High-confidence target genes of chromatin-associated APC increase or decrease in expression following *APC s*ilencing *in vitro* (Column 3 compared to Column 2) as well as in mouse adenomas with activated canonical Wnt signaling (Columns 4 and 5). "*siSCR* fold-change" and "*siAPC* fold-change" refer to the ratio of FPKM from scrambled siRNA-transfected or anti-*APC* siRNA-transfected HCT-116 cells to FPKM from untransfected control cells. "AOM/DSS fold-change" and "*Apc*$^{Min/+}$ fold-change" refer to the average ratio of FPKM from three colon adenomas from either AOM/DSS-treated or *Apc*$^{Min/+}$ mice to their respective non-adenoma colon tissue controls. Column 6 (Total # of peaks) lists for each gene the number of associated genomic sequences enriched by APC ChIP-seq, while the subsets of those peaks that contain at least one TCF7L2 or AP-1 motif are listed in columns 7 and 8.

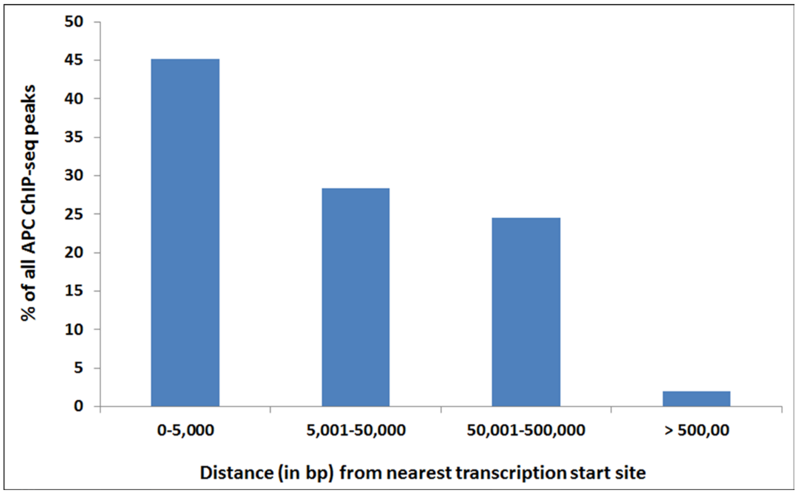

Supplementary Figure 5: Regions enriched by APC ChIP-seq exhibit broad genomic distribution. Anti-APC ChIP-seq peaks exhibited a broad genomic distribution in which more than 50% of enriched genomic sequences were located more than 5-Kb from a transcription start site.

Source of cDNA	*Ctnnb1* Mutation Identified
AOM/DSS-treated mouse adjacent colon tissue	none / wild-type
AOM/DSS-treated mouse colon adenoma 1	D32N
AOM/DSS-treated mouse colon adenoma 2	S33F
AOM/DSS-treated mouse colon adenoma 3	D32N

Supplementary Figure 6: Colon adenomas from AOM/DSS-treated mice encode a degradation-resistant mutant form of β-catenin. Sanger sequencing confirms that colon adenomas from AOM/DSS-treated mice contain activating mutations in the gene (*Ctnnb1*) encoding β-catenin. The presence of this mutation strongly suggests that the adenomas are wild-type for *Apc*, as reported in a published study of the AOM/DSS model [16].

A

Primer Name	Sequence (first binding site underlined in sense strand)
NRF1s	CACTGCGCATGCGCAGCCACTGCGCATGCGCAGCCACTGCGCATGCGCAGC
NRF1as	GATCGCTGCGCATGCGCAGTGGCTGCGCATGCGCAGTGGCTGCGCATGCGCAGTGGTAC
SP1s	ATTCGATCGGGGCGGGGCATTCGATCGGGGCGGGGCATTCGATCGGGGCGGGGC
SP1as	GATCGCCCCGCCCCGATCGAATGCCCCGCCCCGATCGAATGCCCCGCCCCGATCGAATGTAC
AP1s	CGCTTGATGACTCAGCCGGAACGCTTGATGACTCAGCCGGAACGCTTGATGACTCAGCCGGAA
AP1as	GATCTTCCGGCTGAGTCATCAAGCGTTCCGGCTGAGTCATCAAGCGTTCCGGCTGAGTCATCAAGCGGTAC
ZBTB14s	AGGTGCGCGGCCCACAGGTGCGCGGCCCACAGGTGCGCGGCCCAC
ZBTB14as	GATCGTGGGCCGCGCACCTGTGGGCCGCGCACCTGTGGGCCGCGCACCTGTAC
EGR1s	CGCTGCGGGGGCGGAGCGCTGCGGGGGCGGAGCGCTGCGGGGGCGGAG
EGR1as	GATCCTCCGCCCCCGCAGCGCTCCGCCCCCGCAGCGCTCCGCCCCCGCAGCGGTAC
USFs	TCTGGCCCACGTGACCTTCTCTGGCCCACGTGACCTTCTCTGGCCCACGTGACCTTC
USFas	GATCGAAGGTCACGTGGGCCAGAGAAGGTCACGTGGGCCAGAGAAGGTCACGTGGGCCAGAGTAC
E2F3s	CATGGCGCCTCAACATGGCGCCTCAACATGGCGCCTCAA
E2F3as	GATCTTGAGGCGCCATGTTGAGGCGCCATGTTGAGGCGCCATGGTAC
E2F1s	AGTGGCGGGAAGAGTGGCGGGAAGAGTGGCGGGAAG
E2F1as	GATCCTTCCCGCCACTCTTCCCGCCACTCTTCCCGCCACTGTAC

B

Primer Name	Primer Sequence
hsANGPT2chipS	Caacaactggaaggggaaga
hsANGPT2chipAS	Tctgagaaatggtgctgctg
hsFAT1chipS	Gctgcattccgagtttcagt
hsFAT1chipAS	Tgattctgggtcctccctta
hsGPRC5AchipS	Aaaccaaggcattggagatg
hsGPRC5AchipAS	Ggcaatacgtgtttttgctg
KDM6Bchip1s	Cttagccagcacacccactc
KDM6Bchip1as	Gatgctaagaccacgggaag
MACROD1chip1s	Gggcaccctggaacttgtag
MACROD1chip1as	Gctcagccaaccagaaagtt
hsMALLchipS	Cagcaggttggaatgaaggt
hsMALLchipAS	Gttaaaagcggagaccatgc
hsPHLDB2chipS	Agccacaattcctgagcatt
hsPHLDB2chipAS	Aagggtgctggtagacatgg
hsTOM1L2chipS	Gctgctgacccaagttcttt
hsTOM1L2chipAS	Tttccatcctggccctaaat
hsTSPAN5chip3s	cATCAAAGggtctgaaagctg
hsTSPAN5chip3as	Ttttgaagtgggacagagga
NCLchip1s	Cctttcccagcgagacaac
NCLchip1as	Gtatcggccctagtcggagt

Supplementary Figure 7: Primer sets for luciferase cloning and ChIP qPCR. Primer pairs in panel (**A**) were used to generate the firefly luciferase constructs studied in Supplementary Figure 1. Primer pairs in panel (**B**) were used to generate the qPCR data shown in Supplementary Figure 3.
